# Attitudes of university hospital staff towards in-house assisted suicide

**DOI:** 10.1371/journal.pone.0274597

**Published:** 2022-10-27

**Authors:** Claudia Gamondi, Angèle Gayet-Ageron, Gian Domenico Borasio, Samia Hurst, Ralf J. Jox, Bara Ricou

**Affiliations:** 1 Palliative and Supportive Care Service, Lausanne University Hospital and University of Lausanne, Lausanne, Switzerland; 2 Palliative and Supportive Care Clinic, Oncology Institute of Southern Switzerland, Bellinzona, Switzerland; 3 CRC & Division of Clinical-Epidemiology, Department of Health and Community Medicine, University of Geneva & University Hospitals of Geneva, Geneva, Switzerland; 4 Institute for Ethics, History, and the Humanities, Faculty of Medicine, University of Geneva, Geneva, Switzerland; 5 Institute for Humanities in Medicine, Lausanne University Hospital and University of Lausanne, Lausanne, Switzerland; 6 Intensive Care, Department of Acute Care Medicine and Conseil d’éthique Clinique, University Hospitals of Geneva, University of Geneva, Geneva, Switzerland; University of Auckland, NEW ZEALAND

## Abstract

**Objective:**

To investigate staff attitudes toward assisted suicide in the hospital setting in Switzerland.

**Design:**

Cross-sectional study.

**Setting:**

Two University Hospitals in French speaking regions of Switzerland.

**Participants:**

13’834 health care professionals, including all personnel caring for patients, were invited to participate.

**Main outcome measures and other variables:**

Attitudes towards the participation of hospital health care professionals in assisted suicide were investigated with an online questionnaire.

**Results:**

Among all invited professionals, 5’127 responded by filling in the survey at least partially (response rate 37.0%), and 3’683 completed the entire survey (26.6%). 73.0% of participants approved that this practice should be authorized in their hospital and saw more positive than negative effects. 57.6% would consider assisted suicide for themselves. Non-medical professionals were 1.28 to 5.25 times more likely to approve assisted suicide than physicians (p<0.001). 70.7% of respondents indicated that each professional should have the choice of whether to assist in suicide.

**Conclusions:**

This multiprofessional survey sheds light on hospital staff perceptions of assisted suicide happening within hospital walls, which may inform the development of rules considering their wishes but also their reluctances. Further research using a mixed-methods approach could help reach an in-depth understanding of staff’s attitudes and considerations towards assisted suicide practices.

## Introduction

The respect of patients’ autonomy in end-of-life decisions may provoke questions regarding health care professionals’ roles. This paper reports the results of a survey conducted in two university hospitals in Switzerland about staff attitudes towards assisted suicide and its practices within hospital premises. Assisted suicide refers to persons with full decision-making capacity who voluntarily choose to hasten their own death by asking someone else to provide a drug that they will then ingest to end their life. It differs from euthanasia in that the act inducing death must be performed by the person herself [1]. In Switzerland, the penal code prohibits euthanasia, while suicide assistance is prohibited only when it is performed out of selfish motives (e.g., enrichment or other material benefits for the helper) [2]. Whereas Oregon and several other US states have adopted forms of exclusively physician-assisted suicide, the Swiss practice is largely dominated by citizen-directed right-to-die organizations, with limited participation of physicians [3]. Suicide assistance is provided almost exclusively by the volunteers of right-to-die organizations, usually lay people, trained by the organizations themselves without external control on their qualifications.

While a Swiss federal law regulating assisted suicide provision is absent, three French-speaking regions (Vaud, Neuchatel, and Geneva) have enacted legislation regulating assisted suicide in publicly recognized health and long-term care institutions.

Due to the lack of regulatory legislation, the right-to-die organizations set their own rules of conduct, often following informal discussions with local prosecutors. In Switzerland, approximately 15 out of 1,000 deaths result from assisted suicide [4]. Most of them occur at the patient’s home, with 9% of home deaths being certified as assisted suicides [5]. Still, in 2005, the Swiss National Advisory Commission on Biomedical Ethics recommended that every acute care hospital adopt a policy regulating inpatients’ access to assisted suicide within its premises.

In 2006, two Swiss University Hospitals introduced institutional directives specifying the conditions under which assisted suicide may be allowed within the hospital, aiming to ensure that patients with conditions that impeded discharge would be entitled to the same rights as all other citizens [6]. Both policies require that the patients should have decision-making capacity, suffer from a severe medical condition leading eventually to death, and be unable to be discharged home for medical reasons. While hospital professionals are called to evaluate the case, volunteers of the right-to-die associations usually provide the preparation and delivery of the lethal medication to the patient for self-administration.

In Lausanne, there has been a mean of approx. two suicide assistances per year in the last years, with a slightly increasing tendency. In Geneva, there have been 12 requests since 2017, including three from outside Switzerland, which were refused.

To assess the current perspectives of hospital staff regarding assisted suicide happening within hospital premises, a survey was launched in two Swiss French-speaking university hospitals. The primary objective of this study was to evaluate the attitude of the clinical health care professionals as to whether assisted suicide in the hospital should be authorized. The secondary outcomes were the attitudes towards the participation of health care professionals in assisted suicide and towards assisted suicide for themselves.

## Methods

A cross-sectional study was conducted using a self-administered online questionnaire. The instrument was derived from a questionnaire employed in a nationwide survey of general practitioners by the Swiss Academy of Medical Sciences [7]. It was adapted for use by all hospital health care professionals. It consisted of 15 main questions with a total of 80 sub-questions, addressing experiences and practices and personal attitudes concerning assisted suicide. Items on demographic characteristics were also included (S1 File). The questionnaire was converted to an online form using the software REDCap (Vanderbilt University).

The Geneva and Lausanne University Hospitals are tertiary hospitals located in the French-speaking region and are part of the five university hospitals in Switzerland. They have 2’800 and 1’554 beds, respectively.

The target sample consisted of all employees working in patient care or comfort, i.e., physicians, nurses, nurse-assistants, psychologists, social workers, chaplains, physiotherapists, occupational therapists, and dietitians. Employees working in the administration, in the radiology department or exclusively in research were excluded.

The management boards of the two hospitals gave their permission for the study to be conducted. In accordance with Swiss law on research regarding human subjects, the president of the Geneva Cantonal Research Committee for Research waived the need for a full IRB procedure. Participants were fully informed about the aims, background, methods, content, voluntariness, and anonymity of the study and consented by completing and submitting the questionnaire.

All employees have been invited to participate in the survey by personalized e-mails with an online link. REDCap allowed only one single response per participant. After one month, two reminders were automatically sent out to the employees who had not yet responded. The survey was conducted from June 8^th^ to October 8^th^ 2018, in Geneva and from April 4^th^ to August 4^th^ 2019, in Lausanne.

The primary outcome was whether assisted suicide should be authorized in the hospital (yes/no answer). Secondary outcomes were: 1) the attitudes towards the participation of health care professionals in assisted suicide (categorical variable defined by three response options: “professionals must not provide assistance”; “they should have the choice whether to provide assisted suicide”; “they have to comply with a patient’s request for assisted suicide”); 2) the consideration towards assisted suicide for themselves (4-point response options: “no”, “rather no”, “rather yes”, “yes” which were secondly dichotomized as “yes”/”no”). In all cases, the options related to undecided participants were excluded from the analyses.

### Statistical analysis

We summarized continuous variables using descriptive statistics, including number of participants (n), mean, standard deviation (SD), median, minimum, and maximum. For categorical variables, summaries included counts of participants and percentages. We report data of respondents who completed the entire survey (n = 3683) and did not consider those who completed the survey partially. We first compared socio-demographic variables between the two hospitals using either Chi square test for categorical variables or Student’s t-test for continuous variables. We explored the determinants associated with the primary outcome (i.e., favorable attitude toward suicide assistance authorization at the hospital) by performing unconditional logistic regression models. In univariate analyses, we explored gender, age in categories, country where the professional license was obtained (Switzerland vs. other countries), health care profession, professional activity duration, type of clinical specialty, and whether they had a positive attitude towards assisted suicide for themselves. All independent variables associated with the outcome with p<0.25 at univariate or variables that had a negligible proportion of missing data (defined as <5%) were included in multivariable model; we forced gender into the multivariable model. We also used the same regression model to assess the variables associated with the secondary outcome on the consideration toward assisted suicide for themselves. We performed ordinal logistic regression models to evaluate the variables associated with the three ordinal categories for secondary outcome on the general attitude toward the participation of health care professionals in suicide assistance (1 = professionals must not provide assistance, 2 = they should have the choice whether to provide suicide assistance and 3 = they have to comply with a patient’s request for suicide assistance). For both secondary outcomes, we constructed the multivariable models with the same procedure as for the primary outcome. For all models, we report both crude and adjusted odds ratios (ORs) and 95% confidence intervals (95% CI). All analyses were performed using STATA V.IC 16 for Windows (STATA Corp., College Station, Texas, USA). Statistical significance was defined as p<0.05 (two-sided).

## Results

Among the 13’834 health care professionals invited, 5’127 responded by filling in the survey at least partially (response rate 37.0%), and 3’683 completed the entire survey (26.6%). Table 1 shows the respondent characteristics using data from respondents who completed the entire survey. We compared women and men with no missing data as 74% of respondents were women. We found that female participants were younger and more frequently nurses in comparison to men (S1 Table).

**Table 1:** Principal characteristics of the sample (N = 3’683).

Sample characteristics	
Gender, n (%)	
*Male*	955 (25.9)
*Female*	2728 (74.1)
Age categories, n (%)	
*20–39 years*	1746 (47.4)
*40–59 years*	1778 (48.3)
*> = 60 years*	159 (4.3)
Country where professional license was obtained, n (%)	
*Switzerland*	2080 (56.5)
*Other country*	1603 (43.5)
Profession, n (%)	
*Care assistants*	302 (8.2)
*Nurses*	1904 (51.7)
*Physicians*	850 (23.1)
*Physiotherapists*	112 (3.0)
*Occupational therapists (ergotherapists)*	72 (1.9)
*Psychologists*	118 (3.2)
*Chaplains*	16 (0.4)
*Social workers*	33 (0.9)
*Others*	276 (7.5)
Type of clinical specialty, n (%)	
*Medicine*	1577 (42.8)
*Surgery*	684 (18.6)
*Psychiatry*	520 (14.1)
*Pediatrics*	317 (8.6)
*Palliative care*	67 (1.8)
*Other*	514 (14.0)
*Missing*	4 (0.1)
Mean duration of professional activity (since license obtained),	
years (±standard deviation, median, min-max)	16.2 (±10.7, 15, 0–5)
Study site, n (%)	
*Geneva University Hospital*	2269 (61.6)
*Lausanne University Hospital*	1414 (38.4)
Religion, n (%)	
*Catholic*	1336 (36.3)
*Protestant*	432 (11.7)
*Muslim*	82 (2.2)
*Jewish*	14 (0.4)
*Buddhist*	25 (0.7)
*Hindu*	7 (0.2)
*Other religion*	1119 (3.2)
*I do not have a religion*	1341 (36.4)
*No answer*	327 (8.9)

Participants were mostly women, reflecting the study population. Nearly half were younger than 40 years, and more than half obtained their professional license in Switzerland. Over 60% were nurses or health care assistants. Almost a quarter were physicians, more frequently internists (43%), and with a mean duration of professional activity of 17 years.

Among the respondents, 1’791 (48.6%) reported past experience with requests for suicide assistance in the context of their professional activity in the hospital; 442 (12.0%) indicated having been asked 6 times or more.

### 1. Allowing assisted suicide inside hospital as a professional

**Fig 1:**
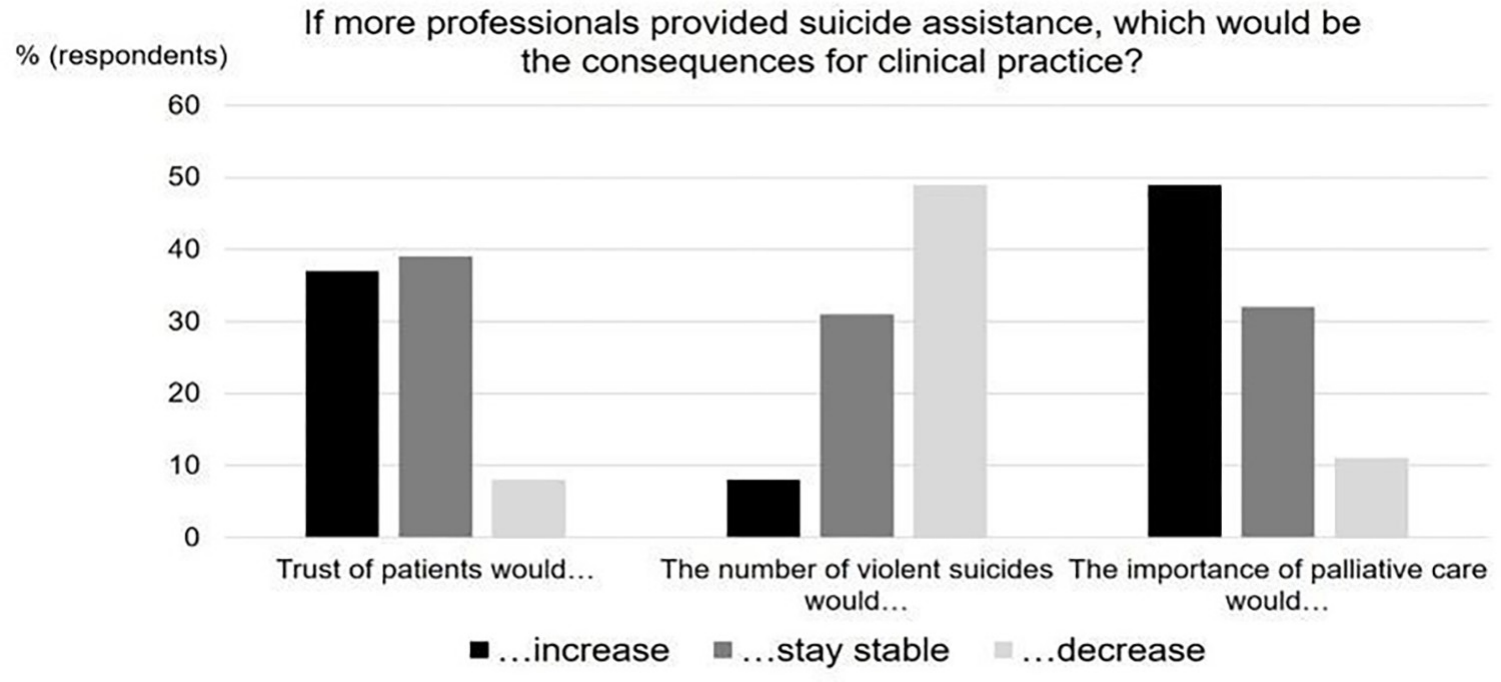
Expected consequences of more professionals providing suicide assistance. Participants did not think that patient trust would decrease. They mostly assumed that violent suicides would decrease and that the importance of palliative care would increase.

Overall, 73.0% (n = 2687) of respondents expressed the view that assisted suicide should be authorized in their hospital; 13.7% (n = 505) were against it and 13.3% (n = 491) were undecided. In decreasing frequency, respondents (n = 3178) were favorable towards permitting the following persons to perform suicide assistance: qualified hospital physicians (84.5%, n = 2684), assisted-suicide organizations (82.4%, n = 2618), qualified hospital nurses (65.0%, n = 2065), qualified physicians from outside the hospital (63.2%, n = 2009), and qualified nurses from outside the hospital (46.1%, n = 1466). The participants’ main motivation was that allowing health care professionals to assist suicide would bring rather positive consequences (see Fig 1). Participants did not think that patient trust would decrease. They mostly assumed that violent suicides would decrease and that the importance of palliative care would increase.

The factors associated with the attitudes toward assisted suicide inside the hospital are summarized in Table 2.

**Table 2:** Factors associated with favoring the authorization of assisted suicide inside the hospital (univariate and multivariable analyses).

	Univariate			Multivariate		
Variables, (n observations available, univariate)	Odds ratio	IC95%	p-value	Odds ratio	IC95%	p-value
Gender (n = 3’192)			0.050			0.313
Male	1.00	0.99–1.52		1.00	-	
Female	1.23			0.86	0.64–1.16	
Age (n = 1’951)			0.396	-	-	-
20–39 years	1.00	-	-			
40–59 years	0.87	0.67–1.14	0.317			
> = 60 years	0.71	0.40–1.26	0.245			
Country of training (n = 3’192)			0.222			0.013
Switzerland	1.00	-		1.00	-	
Other country	0.89	0.73–1.07		0.70	0.53–0.93	
Profession (n = 3’192)			<0.001			<0.001
Physicians	1.00	-	-	1.00	-	-
Nurses	2.10	1.69–2.61	<0.001	1.32	0.96–1.80	0.083
Therapists/Psychologists	5.25	3.09–8.94	<0.001	4.80	2.43–9.50	<0.001
Care assistants	1.28	0.90–1.82	0.173	0.92	0.55–1.55	0.757
Others (chaplains, social workers, others)	2.96	1.93–4.52	<0.001	1.51	0.87–2.61	0.146
Duration of professional activity (n = 3’192)			0.146	-	-	-
<5 years	1.00	-	-			
5–10	0.67	0.48–0.94	0.022			
10–20	0.82	0.59–1.13	0.217			
20–30	0.9	0.63–1.26	0.520			
> = 30 years	0.74	0.52–1.06	0.100			
Type of clinical specialty (n = 3’285)			<0.001			0.032
Medicine	1.00	-	-	1.00	-	
Surgery	1.12	0.86–1.46	0.416	1.04	0.73–1.49	0.828
Psychiatry	0.83	0.63–1.09	0.180	0.70	0.48–1.03	0.067
Palliative care	0.42	0.24–0.75	0.003	0.45	0.20–1.04	0.061
Paediatrics	1.45	0.98–2.14	0.062	1.39	0.81–2.39	0.235
Others	1.46	1.06–2.02	0.020	1.37	0.88–2.13	0.163
Positive toward assisted suicide for oneself (n = 2’655)			<0.001	26.12	19.65–34.73	<0.001
No	1.00	-				
Yes	27.19	20.64–35.80				
Religion (n = 3’192)			<0.001			0.025
Protestant	1.00	-	-	1.00	-	-
Catholic	1.39	1.04–1.84	0.025	1.36	0.91–2.02	0.134
Other	1.87	1.41–2.47	<0.001	1	1.14–2.47	0.008

In univariate analysis, gender, professional category, type of clinical specialty, having a positive attitude toward assisted suicide for oneself, and religion were all significantly associated with favoring the authorization of assisted suicide inside the hospital. We kept the following variables in a multivariable model: gender, country of training, professional category, type of clinical specialty, positive toward assisted suicide for oneself, and religion.

In multivariable analysis, the variables independently associated with favoring the authorization of assisted suicide inside the hospital were: training in Switzerland vs. in another country (positive association), profession (being a therapist provided a higher odds for favoring the authorization of assisted suicide inside the hospital compared to physician), being positive toward assisted suicide for oneself (positive association), and religion (‘other religion’ provided higher odds for favoring the authorization of assisted suicide inside the hospital compared to protestant).

### 2. Assisted suicide for themselves

Participants were also asked to indicate whether they would consider assisted suicide for themselves: 1199 answered “yes” (32.6%) or 922 “rather yes” (25.0%), 362 responded “rather no” (9.8%) or 505 “no” (13. 7%), 695 (18.9%) were undecided or did not want to respond and were excluded from the analysis. The S2 Table summarizes the results of univariate and multivariate analyses of the variables associated with a favorable attitude toward assisted suicide for themselves. In the multivariable model, only profession (compared to physicians, all other professions provided higher odds towards a favorable attitude toward assisted suicide for themselves), type of clinical specialty (pediatrics and other specialty provided higher odds compared to medicine) and religion (catholic and other religion provided higher odds compared to protestant) were significantly associated with the outcome.

### 3. Participation in assisted suicide practices

**Fig 2:**
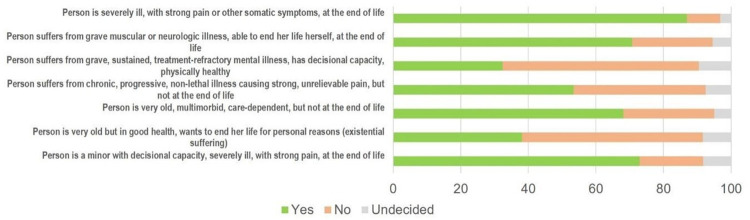
Attitudes of health care professionals towards the legitimacy of assisted suicide in different patient populations.

When participants were asked about their opinion concerning health care professionals’ participation in assisted suicide, they were asked to abstract from their personal readiness to deliver such assistance: 70.7% (n = 2603) stated that each professional should have the choice whether or not to assist suicide, 19.8% (n = 728) responded that professionals have to comply with a patient’s request for assisted suicide, and 7.2% (n = 267) held that they must not assist in suicide. Their general attitude about the legitimacy of assisted suicide given by a physician varied, however, depending on the different underlying health conditions of the patient (Fig 2). A majority of respondents agreed with assisted suicide in patients with severe somatic illness and suffering at the end of life, including minors with decision-making capacity, as well as in multimorbid geriatric patients. Opinions were divided concerning patients with non-lethal illnesses and pain. Most respondents did not favor assisted suicide for patients with mental illness or older people in good health.

**Fig 3:**
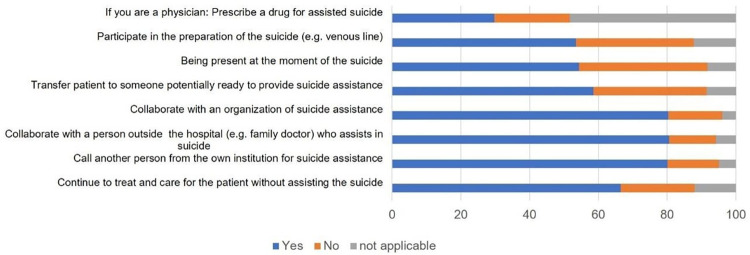
Readiness to participate in assisted suicide.

Participants were also asked how they would react if confronted with a request for assisted suicide. Almost all respondents would inform the patient about prognosis and alternative care options (97.1%, n = 3576), examine whether the patient has decisional capacity (97.9%, n = 3605), investigate whether the wish to die is well reflected, voluntary and persistent (97.5%, n = 3591) explore the motivations of the wish to die (96.6%, n = 3555), and request a palliative care consultation (93.0%, n = 3426). The core action of suicide assistance, consisting in prescribing or handing over the lethal drug to the patient, was imaginable for 60.3% (n = 2222) of the participants, while 31.0% (n = 1140) could not envisage it and 8.7% (n = 320) were undecided. When only the answers of physicians were considered, 58.5% (n = 497) would be ready to prescribe a lethal medication. The readiness of all participants to participate in diverse manners in suicide assistance is shown in Fig 3. Most participants would be willing to collaborate with a right-to-die association that performs the assistance. About half would be willing to participate actively, e.g., by placing a venous line or (for physicians) by prescribing the lethal drug and to be present at the moment of suicide. Two-thirds would agree to continue treating the patients without assisting in the suicide.

At univariate analyses (S3 Table, left panel), the variables associated with greater odds for a positive attitude toward the participation of health care professionals in assisted suicide were: other health care professions than physicians, other types of clinical specialty than medicine, catholic or other religion than protestant. In multivariable analysis (S3 Table, right panel), greater odds for a positive general attitude toward the participation of healthcare professionals in assisted suicide were found for: all other healthcare professions (compared to physicians), and for professionals who declared having another/no religion (compared to catholic). The odds were lower for professionals working in palliative care compared to the medicine sector.

## Discussion

To our knowledge, this is the first European study investigating the attitudes of a multi-professional hospital staff on suicide assistance in their institutions. The salient findings of this study were that the great majority of the professionals in these two Swiss university hospitals favored assisted suicide occurring inside their institution, that healthcare professionals should have the choice to participate on a voluntary basis in assisted suicide practices, and that a significant proportion was ready to assist patients themselves. The professionals identified the hospital physicians as the most appropriate person to help the patients. This is remarkably sustained by the fact that more than half of the hospital physicians were willing to assist patients in committing suicide inside the institution. Yet, this is in contrast to the current procedure that relies on external right-to-die organizations to assist hospitalized patients to die by assisted suicide. Health care professionals were frequently confronted with patients’ requests for assisted suicide. Our findings were consistent with the worldwide increased recognition of individual autonomy, the global rise in societal acceptance of assisted dying, and the incremental legalization of assisted suicide in more and more European and US jurisdictions over the last decade [3, 8].

In jurisdictions that have recently legalized assisted dying, there are ongoing studies aiming to describe views and experiences of hospital clinical staff about the actual or upcoming impact of providing assisted dying within hospital wards [9–13]. However, none reported the personal insight of the healthcare providers nor the motivations leading to their attitudes. Our study showed that despite the restrictive institutional policies regarding assisted suicide, the participants seemed quite open to a more active involvement. Although participants’ general attitudes favored assisted suicide inside the hospital, the study revealed substantial heterogeneity in hospital staff’s attitudes. Indeed, compared to nurses, physicians were less favorable to this practice, palliative care physicians and psychiatrists less so than internists; non-Swiss and professionals of protestant confession were also less favorable to this practice. While the general population predominantly approves of the availability of assisted suicide in Western countries, [3, 14], medical organizations are much more critical, specifically some palliative care organizations [1, 15]. In Switzerland, while a national survey conducted in 2015 has shown that 70% of physicians agree with the availability of assisted suicide [7], in 2019, the Swiss medical association has refused to adopt an end-of-life guideline of the Swiss Academy of Medical Sciences, considering the proposed recommendations on assisted suicide as being too permissive (they were finally adopted, after revision and clarification, in 2022). The rather critical stance of physicians, as expressed by their professional body, may be partly due to their closer participation and responsibility in suicide assistance and partly to traditional views of the professional code of practice for physicians. Such discussion contrasts with the practice of assisted suicide performed worldwide by physicians [16]. More research is needed to better understand the physicians’ ambivalence towards assisted dying and to inform policy makers on this point.

Our data also confirm the well-known reticence of many palliative care physicians concerning assisted dying [17]. Swiss palliative care physicians have historically taken a critical stance toward suicide assistance. However, this has evolved in recent years towards a more neutral view, as evidenced also by the research conducted in this field e.g., by several of this paper’s authors [2, 5, 14, 18].

The most important factor associated with the propensity to favor assisted suicide was the fact that the persons would consider assisted suicide for themselves. This finding is of key importance since such thoughts usually reflect personal experience about the significance of life, disease, suffering, and death. Some reports in the literature suggest that physicians and nurses may be motivated to provide assisted dying, especially when they are familiar with the act [19]. Swiss palliative care physicians have also expressed the need for better training on how to deal with requests for suicide assistance, more professional exchange among themselves and with right-to-die organizations, as well as an open debate on assisted suicide in hospitals [20]. In the Netherlands, nurses in academic hospitals think that a nurse should also be allowed to administer the lethal drugs [21]. In the Canadian assisted dying practice, nurses are involved in 10% of the cases [22]. As nurses are usually engaged in communication and decision making about suicide assistance, and more countries introduce advance practice nurses, it remains an open question what role nurses (or other non-physician professionals) should play in assisted dying [23].

Religiosity also seems to play a role, as protestant participants were less likely than those of other religions to favor assisted suicide. In a survey of 135 Israeli hospital physicians [24], 54% favored legalization of euthanasia (more than in most other countries). This opinion was significantly negatively correlated with their level of religiosity. A similar correlation with religiosity was found in a sample of older adults in New Zealand, where a large majority of respondents did not support the legalization of assisted dying [25].

The ample spectrum of criteria that participants identified as necessary to accept that assisted suicide may happen in hospital premises underlines the diverse sensitivities of the healthcare professionals. Our findings might help politicians and directors of institutions to establish rules that can be acceptable for the majority of the professionals and respectful of minorities.

### Strengths and limitations of the study

The major strength of this study is its originality since no other study explored the attitudes of such a wide range of healthcare professionals about assisted suicide inside the hospital. The response rate was relatively good considering the conflict of interest with which the healthcare professionals might have been confronted between their duty to preserve life and their willingness to help patients, and that could have discouraged participation. Other strengths of the study include its multiprofessional character and the representativity of the study population.

This survey also has several limitations. First, the results might not be generalizable to other regions in Switzerland or to non-university hospitals, nor to other countries with different or no legislation around assisted suicide. Second, as with most survey studies, there is the risk of a social desirability bias, which we strived to mitigate by the anonymous nature of the survey and its online format. Also, a sampling bias cannot be ruled out: professionals with strong opinions may have been more likely to participate, but this would likely apply to both ends of the spectrum.

## Conclusions

Our data show that a large majority of the surveyed hospital staff are ready to accept assisted suicide in their institution. The results shed light on the perception of the hospital staff, allowing the development of rules considering their wishes but also their reluctances. Hospital boards should clarify the voluntary role of healthcare professionals in assisted suicide practices and provide guidelines, educational programs for all staff, and support professionals with debriefings during and after the procedure. Particular attention should be given to the communication between patients, relatives, and health care professionals on these issues, as their views might differ strongly on the subject.

In future research, a mixed-methods approach may help attain a more in-depth understanding of hospital staff reasoning and considerations towards assisted dying.

## Supporting information

S1 Table Comparison of female (n = 1691) to male responders (n = 587) with no missing data for the three variables profession, age category, and duration of professional experience. (DOCX)

S2 Table Factors associated with positive general attitude toward the assisted suicide for oneself (univariate and multivariable analyses). (DOCX)

S3 Table Factors associated with a positive general attitude toward the participation of health care professionals in assisted suicide (univariate and multivariable analyses). (DOCX)

S4 Table Professions’ distribution of the participants that reported experience with requests for suicide assistance in the context of their professional activity in the hospital. (n = 1’850 due to missing data for some sociodemographic variables).
(DOCX)

S1 File Forward-backward translation in English of the original questionnaire in French. (DOCX)
