# Supplementary material for: Attitudes of university hospital staff towards in-house assisted suicide
Source: PLoS One. 2022 Oct 27;17(10):e0274597. doi: 10.1371/journal.pone.0274597 (PMC9612505; doi:10.1371/journal.pone.0274597)
Supplement: S1 Table — (DOCX) [file pone.0274597.s001.docx]

**Supplementary table 1.** Comparison of female (n=1691) to male responders (n=587) with no missing data for the three variables profession,

age category, and duration of professional experience.

|  | **Men (n=587)** | **Women (n=1691)** | **p-value** |
| --- | --- | --- | --- |
| Profession, n (%)  Nurses  MD  Physio/ergo/psycho  Care assistants  Other | 242 (41.2)  229 (39.0)  31 (5.3)  28 (4.8)  57 (9.7) | 931 (55.1)  247 (14.6)  125 (7.4)  166 (9.8)  222 (13.1) | <0.001 |
| Age category, n (%)  20-39  40-59  >=60 years | 223 (38.0)  319 (54.3)  45 (7.7) | 685 (40.5)  943 (55.8)  63 (3.7) | 0.001 |
| Duration of prof, n (%)  <5  5-20  20-30  >=30 | 73 (12.4)  261 (44.5)  137 (23.3)  116 (19.8) | 222 (13.1)  707 (41.8)  404 (23.9)  358 (21.2) | 0.716 |

Female responders were more frequently nurses (p<0.001), less frequently MD, and more frequently care assistants. They were younger than male responders (p=0.001), but they had no different duration of professional activity (p=0.716)
