# Supplementary material for: Attitudes of university hospital staff towards in-house assisted suicide
Source: PLoS One. 2022 Oct 27;17(10):e0274597. doi: 10.1371/journal.pone.0274597 (PMC9612505; doi:10.1371/journal.pone.0274597)
Supplement: S2 Table — (DOCX) [file pone.0274597.s002.docx]

**Supplementary table 2.** Factors associated with positive general attitude toward the assisted suicide for oneself (univariate and multivariable analyses).

|  | **Univariate** | | | **Multivariable** | | |
| --- | --- | --- | --- | --- | --- | --- |
| **Variables (n observations available, univariate)** | **Odds ratio** | **IC95%** | **p-value** | **Odds ratio** | **IC95%** | **p-value** |
| Gender (n=2’988)  Male  Female | 1.00  1.15 | -  0.97-1.37 | 0.111 | 1.00  0.96 | -  0.79-1.16 | 0.656 |
| Age (n=1’845)  20-39 years  40-59 years  >=60 years | 1.00  1.07  0.68 | -  0.86-1.33  0.43-1.08 | 0.147  -  0.543  0.105 | - | - | - |
| Country of training (n=2’988)  Switzerland  Other country | 1.00  0.84 | -  0.72-0.99 | 0.036 | 1.00  0.96 | -  0.80-1.15 | 0.671 |
| Profession (n=2’988)  Physicians  Nurses  Therapists/Psychologists  Care assistants  Others (chaplains, social workers, social workers, others) | 1.00  2.40  2.40  1.64  2.34 | -  1.98-2.90  1.72-3.35  1.19-2.24  1.70-3.21 | <0.001  -  <0.001  <0.001  0.002  <0.001 | 1.00  2.37  2.56  1.66  2.10 | -  1.93-2.92  1.82-3.61  1.19-2.30  1.50-2.93 | <0.001  -  <0.001  <0.001  0.003  <0.001 |
| Duration of professional activity (n=2’988)  <5 years  5-10  10-20  20-30  >=30 years | 1.00  0.97  1.14  1.39  1.19 | -  0.74-1.28  0.88-1.47  1.06-1.83  0.90-1.58 | 0.053  -  0.840  0.310  0.019  0.223 | 1.00  1.08  1.14  1.38  1.14 | -  0.81-1.44  0.87-1.48  1.03-1.84  0.85-1.54 | 0.243  -  0.588  0.351  0.031  0.388 |
| Type of clinical specialty (n=2’985)  Medicine  Surgery  Psychiatry  Palliative care  Pediatrics  Others | 1.00  1.13  0.91  0.70  1.36  1.54 | -  0.91-1.41  0.72-1.15  0.40-1.22  0.99-1.86  1.19-1.99 | 0.002  -  0.264  0.432  0.207  0.053  0.001 | 1.00  1.11  0.85  0.72  1.40  1.44 | -  0.88-1.39  0.67-1.09  0.41-1.29  1.02-1.94  1.10-1.90 | 0.006  -  0.369  0.206  0.272  0.039  0.008 |
| Religion (n=2’988)  Protestant  Catholic  Other/no | 1.00  1.58  2.18 | -  1.24-2.02  1.72-2.77 | <0.001  -  <0.001  <0.001 | 1.00  1.48  2.21 | -  1.14-1.91  1.72-2.84 | <0.001  -  0.003  <0.001 |
