# Supplementary material for: Attitudes of university hospital staff towards in-house assisted suicide
Source: PLoS One. 2022 Oct 27;17(10):e0274597. doi: 10.1371/journal.pone.0274597 (PMC9612505; doi:10.1371/journal.pone.0274597)
Supplement: S3 Table — (DOCX) [file pone.0274597.s003.docx]

**Supplementary table 3.** Factors associated with a positive general attitude toward the participation of health care professionals in assisted suicide (univariate and multivariable analyses).

|  | **Univariate** | | | **Multivariable** | | |  |
| --- | --- | --- | --- | --- | --- | --- | --- |
| **Variables (n observations available, univariate)** | **Odds ratio** | **IC95%** | **p-value** | **Odds ratio** | **IC95%** | **p-value** | |
| Gender (n=3’598)  Male  Female | 1.00  1.16 | -  0.98-1.37 | 0.079 | 1.00  0.99 | -  0.83-1.18 | 0.929 | |
| Age (n=2’213)  20-39 years  40-59 years  >=60 years | 1.00  0.89  1.26 | -  0.74-1.08  0.82-1.94 | 0.184  -  0.231  0.293 | - | - | - | |
| Country of training (n=3’598)  Switzerland  Other country | 1.00  0.94 | -  0.81-1.08 | 0.380 | 1.00  0.92 | -  0.78-1.08 | 0.298 | |
| Profession (n=3’598)  Physicians  Nurses  Therapists/Psychologists  Care assistants  Others (chaplains, social workers, social workers, others) | 1.00  1.97  3.02  1.95  3.42 | -  1.63-2.38  2.26-4.04  1.43-2.67  2.58-4.54 | <0.001  -  <0.001  <0.001  <0.001  <0.001 | 1.00  2.01  3.25  1.99  3.38 | -  1.64-2.47  2.41-4.37  1.44-2.74  2.51-4.56 | <0.001  -  <0.001  <0.001  0.002  <0.001 | |
| Duration of professional activity (n=3’722)  <5 years  5-10  10-20  20-30  >=30 years | 1.00  0.94  0.95  0.80  0.93 | -  0.73-1.21  0.75-1.19  0.63-1.03  0.72-1.20 | 0.431  -  0.606  0.654  0.081  0.585 | 1.00  1.05  0.94  0.77  0.90 | -  0.81-1.36  0.74-1.19  0.59-0.99  0.68-1.17 | 0.111  -  0.730  0.598  0.045  0.425 | |
| Type of clinical specialty (n=3’595)  Medicine  Surgery  Psychiatry  Palliative care  Paediatrics  Others | 1.00  1.14  0.85  0.53  0.94  1.33 | -  0.93-1.39  0.68-1.06  0.30-0.95  0.72-1.23  1.06-1.65 | 0.003  -  0.212  0.148  0.034  0.650  0.012 | 1.00  1.09  0.81  0.54  0.97  1.19 | -  0.89-1.34  0.64-1.02  0.30-0.96  0.74-1.28  0.95-1.50 | 0.026  -  0.403  0.070  0.037  0.840  0.131 | |
| Religion (n=3’598)  Catholic  Protestant  Other/no | 1.00  0.80  1.27 | -  0.63-1.03  1.08-1.48 | <0.001  -  0.086  0.003 | 1.00  0.86  1.29 | -  0.67-1.11  1.10-1.52 | <0.001  -  0.254  0.002 | |

In univariate analysis, the variables significantly associated with higher odds for positive general attitude toward the participation of health care professionals in assisted suicide were: non-physician profession, type of clinical speciality, and religion other than catholic or protestant. In multivariable analysis, the variables that stayed or became independently associated with higher odds for a positive general attitude toward the participation of health care professionals in assisted suicide were: professional category, type of clinical speciality, and religion.
