# Supplementary material for: Attitudes of university hospital staff towards in-house assisted suicide
Source: PLoS One. 2022 Oct 27;17(10):e0274597. doi: 10.1371/journal.pone.0274597 (PMC9612505; doi:10.1371/journal.pone.0274597)
Supplement: S4 Table — (n = 1’850 due to missing data for some sociodemographic variables). (DOCX) [file pone.0274597.s004.docx]

**Supplementary table 4.** Professions’ distribution of the participants that reported experience with requests for suicide assistance in the context of their professional activity in the hospital (n=1’850 due to missing data for some sociodemographic variables)

| Nurses | 1’044 | 56.4% |
| --- | --- | --- |
| Physicians | 414 | 22.4% |
| Physio/ergo-therapists | 131 | 7.1% |
| Care assistants | 184 | 9.9% |
| Other professions | 77 | 4.2% |
