# Supplementary material for: Attitudes of university hospital staff towards in-house assisted suicide
Source: PLoS One. 2022 Oct 27;17(10):e0274597. doi: 10.1371/journal.pone.0274597 (PMC9612505; doi:10.1371/journal.pone.0274597)
Supplement: S1 File — (DOCX) [file pone.0274597.s005.docx]

**Experiences and attitudes towards**

**assisted suicide**

Dear colleagues,

The objective of this questionnaire is to find out the opinions of health professionals at the University hospitals of Geneva (HUG) and Lausanne (CHUV) concerning assisted suicide. Health professionals include all those involved in the care of hospitalized patients in the services.

If you agree to answer this questionnaire, you do so completely anonymously. We will have no way of checking your identity.

Assisted suicide (synonym: aid in dying) refers to all activities that contribute to a person wishing to die to be able to kill him/herself by an act that he/she carries out him/herself. This applies in particular to the prescription or supply of a drug for suicide, such as sodium pentobarbital (*Natrium pentobarbital*), which is only supplied on prescription.

Throughout the text, the male gender has been used to ease reading. It read always for all genders.

This questionnaire is the result of a reflection by the Clinical Ethics Commissions of the HUG and CHUV. The survey is carried out in agreement with the Medical Directorates of both hospitals.

We thank you very much in advance for your participation.

To begin with, in which hospital do you work?

- At the HUG
- At the CHUV

**1. General attitude towards the participation of health professionals in assisted suicide**

Whether or not you are personally willing to provide assisted suicide, what is your **fundamental** attitude towards the involvement of health professionals in assisted suicide? (Please click the answer that suits you)

| I agree |  |
| --- | --- |
| ⃝ | 1. Health professionals **should not** provide assisted suicide, even when permitted by law |
| ⃝ | 2. Health professionals **should have the choice** to provide assisted suicide or not, when permitted by law |
| ⃝ | 3. Health care professionals **must** fulfil the wishes of patients who request assisted suicide |
| ⃝ | 4. I have no opinion |

**2. General attitude towards assisted suicide in specific situations1**

Whether or not you are personally prepared to provide assisted suicide, do you think it is *in principle defensible* for a doctor to prescribe or give a drug to a patient in the categories of situations described below, enabling the patient to end his or her life?

*Assume in all these situations that the patient has his decision-making capacity*.

|  | Yes | Rather yes | Rather no | No | I don't know |
| --- | --- | --- | --- | --- | --- |
| 1. The person who wishes to end his life is seriously ill, suffering from severe pain or other serious somatic symptoms and is at the **end of his life**, i.e. in your experience it is foreseeable that he will die within a few days or weeks. | ⬜ | ⬜ | ⬜ | ⬜ | ⬜ |
| 1. The person who wishes to end his life suffers from a **serious** **muscular or neurological disease** (e.g. amyotrophic lateral sclerosis), but is still able to end his life. He is **not** at the end of his life. | ⬜ | ⬜ | ⬜ | ⬜ | ⬜ |
| 1. The person who wishes to end his life suffers from a **serious mental illness** (e.g. depression) that is long lasting and resistant to therapy, but has his decision-making capacity. The person is physically healthy. | ⬜ | ⬜ | ⬜ | ⬜ | ⬜ |
| 1. The person who wishes to end his life has a **chronic**, progressive, non-mortal **disease** (e.g. polyarthritis) which causes him very severe pain that cannot be alleviated. He is **not** at the end of his/her life. | ⬜ | ⬜ | ⬜ | ⬜ | ⬜ |
| 1. The person who wishes to end his life is very old, has   **polymorbidities** and he is **dependent** on care. He is **not** at the end of his life. | ⬜ | ⬜ | ⬜ | ⬜ | ⬜ |
| 1. The person who wishes to end his life is in good health and very old; he wishes to commit suicide for personal reasons (existential suffering). | ⬜ | ⬜ | ⬜ | ⬜ | ⬜ |
| 1. The person who wishes to end his life is seriously ill, in great pain and at the end of life. He is under 18, but has his capacity of discernment. | ⬜ | ⬜ | ⬜ | ⬜ | ⬜ |

**3. Personal willingness to provide assisted suicide**

Can you think of situations in which you **personally** would be ready, as a health professional, to prescribe or give a medicine to a **person who has her decision making capacity**, allowing them to end their life?

| Yes | Rather yes | Rather no | No | I don't know |
| --- | --- | --- | --- | --- |
| ⬜ | ⬜ | ⬜ | ⬜ | ⬜ |

**4. How to proceed with patients who express the will to commit suicide**

What would you do if a patient came to you with the wish to obtain assisted suicide?

*Please answer each line*

| Yes | No |  |
| --- | --- | --- |
| ⬜ | ⬜ | 1. Inform and advise the person about the prospects for recovery and other medical, care or support options that could alleviate their suffering |
| ⬜ | ⬜ | 1. Request a palliative care consultation |
| ⬜ | ⬜ | 1. Explore the motivations of the death wish in an interdisciplinary way |
| ⬜ | ⬜ | 1. Try to dissuade the person from committing suicide |
| ⬜ | ⬜ | 1. Investigate whether the person has his decision making capacity |
| ⬜ | ⬜ | 1. Assess whether the desire to die is deeply thought, it doesn’t result of external pressures and it is persistent |
| ⬜ | ⬜ | 1. Consider whether the person is at the end of life, i.e. according to your experience, his death is foreseeable within a few days or weeks. |
| ⬜ | ⬜ | 1. Make sure that the requirements are reviewed by a third party |
| ⬜ | ⬜ | 1. Refer the person to another physician who may be willing to provide assisted suicide |
| ⬜ | ⬜ | 1. Refuse further care or ongoing treatment? |

**If you can imagine** situations where you think the conditions for an assisted suicide are fulfilled: go to **Question 5**

**If you cannot imagine** any situation fulfilling the conditions for assisted suicide: go to **Question 6**

**5. Personal attitude if you feel that the conditions for assisted suicide are fulfilled in a specific case**

What would you be ready to do if, in a specific case, all the conditions for assisted suicide were met?

*Please answer each line.*

| Yes | No | Not relevant |  |
| --- | --- | --- | --- |
| ⬜ | ⬜ | ⬜ | 1. If you are a doctor: issue the prescription for a life-ending drug or hand over the drug. |
| ⬜ | ⬜ |  | 1. Participate in the preparation of the suicide, e.g. by placing or leaving a venous line, or by giving specific indications for the suicide |
| ⬜ | ⬜ |  | 1. Be present at the moment of suicide until death occurs |
| ⬜ | ⬜ |  | 1. Refer the person to someone else who may be willing to provide assisted suicide |
| ⬜ | ⬜ |  | 1. Collaborate with an organization providing assisted suicide |
| ⬜ | ⬜ |  | 1. Collaborate with a person outside the hospital, such as the general practitioner, who will assist the patient |
| ⬜ | ⬜ |  | 1. Contact a person in the institution for assisted suicide |
| ⬜ | ⬜ |  | 1. Continue to treat or care for the patient without participating in the suicide |

**6. Frequency of requests for assisted suicide**

How many patients have ever seriously asked you for assisted suicide?

*A rough indication is sufficient.*

|  | Number of patients |
| --- | --- |
| As part of your practice within the HUG/CHUV : |  |
| Outside your work within the HUG / CHUV : |  |

**7. Duties of health professionals in relation to the wish for assisted suicide**

In your opinion, **in relation to your own profession**, what status should have the actions listed in the table below?

|  | It is not a professional duty and should be **prohibited** for health care professionals | It is not a professional duty but it is exceptionally permitted as a personal moral decision | It is a duty that the professional **can offer** voluntarily | It is a duty that the professional is **bound** to propose and, when appropriate, to perform | Doesn't apply to my profession |
| --- | --- | --- | --- | --- | --- |
| 1. Check whether the conditions for assisted suicide are met | ⬜ | ⬜ | ⬜ | ⬜ | ⬜ |
| 1. Discuss with the patient the advantages and disadvantages of assisted suicide compared to other options | ⬜ | ⬜ | ⬜ | ⬜ | ⬜ |
| 1. Help the patient to contact one of the organizations providing assisted suicide | ⬜ | ⬜ | ⬜ | ⬜ | ⬜ |
| 1. Provide ongoing support to the patient | ⬜ | ⬜ | ⬜ | ⬜ | ⬜ |
| 1. Help family members understand the patient's wish for assisted suicide | ⬜ | ⬜ | ⬜ | ⬜ | ⬜ |
| 1. Meet with the staff of one of the organizations providing assisted suicide at the request of a patient | ⬜ | ⬜ | ⬜ | ⬜ | ⬜ |
| 1. Write the diagnostic report required by the organizations providing assisted suicide | ⬜ | ⬜ | ⬜ | ⬜ | ⬜ |
| 1. Prescribing or providing a drug to end life | ⬜ | ⬜ | ⬜ | ⬜ | ⬜ |
| 1. Participate in the preparation of the suicide, e.g. by placing or leaving a venous line, or by giving specific indications for the suicide | ⬜ | ⬜ | ⬜ | ⬜ | ⬜ |
| 1. Be present at the moment of suicide until death occurs | ⬜ | ⬜ | ⬜ | ⬜ | ⬜ |

**8. Assessment of current ethical requirements for assisted suicide**

Do you think that the following conditions must be met for assisted suicide?

|  | Yes | Rather yes | Rather  no | No | I don't know |
| --- | --- | --- | --- | --- | --- |
| 1. The illness justifies the assumption that the patient is at the end of life, i.e. that in your experience, it is foreseeable that he will die within a few days or weeks or months. | ⬜ | ⬜ | ⬜ | ⬜ | ⬜ |
| 2. A second opinion to assess the decision-making capacity has been requested. | ⬜ | ⬜ | ⬜ | ⬜ | ⬜ |
| 3. A second opinion to assess the wish to die has been requested. | ⬜ | ⬜ | ⬜ | ⬜ | ⬜ |
| 4. The second opinion may be given by a person who is not a physician. | ⬜ | ⬜ | ⬜ | ⬜ | ⬜ |

**9a. Consequences of the spread of assisted suicide**

In your opinion, what would be the consequences for daily clinical practice if more professionals were to provide assisted suicide?

|  | certainly  increase | rather increase | remain stable | rather  decrease | certainly  decrease | I don't know |
| --- | --- | --- | --- | --- | --- | --- |
| 1. Patients' trust in them will... | ⬜ | ⬜ | ⬜ | ⬜ | ⬜ | ⬜ |
| 1. The number of violent (unassisted) suicides will... | ⬜ | ⬜ | ⬜ | ⬜ | ⬜ | ⬜ |
| 1. The relevance of palliative medicine will... | ⬜ | ⬜ | ⬜ | ⬜ | ⬜ | ⬜ |

**9b. Consequences of the spread of assisted suicide (second part)**

The Swiss Academy of Medical Sciences (SAMS) has recently relaxed its requirements for assisted suicide. Proximity of death is no longer considered as a requirement, but rather "intolerable suffering" due to the symptoms of an incurable disease or infirmity. The Federation of Swiss Physicians (FMH) did not adhere to these requirements because of the "too indeterminate nature" of this criterion.

|  | Not at all | Rather no | Rather yes | Totally | I don't know |
| --- | --- | --- | --- | --- | --- |
| Do you agree with the introduction of more flexible criteria for assisted suicide proposed by the SAMS? | ⬜ | ⬜ | ⬜ | ⬜ | ⬜ |

**9c. Consequences of the spread of assisted suicide (third part)**

What do you think the consequences would be for everyday clinical practice if the relaxed requirements of the SAMS for assisted suicide were applied in hospitals?

|  | certainly  increase | rather  increase | remain  stable | rather  decrease | certainly decrease | I don't know |
| --- | --- | --- | --- | --- | --- | --- |
| Patients’ satisfaction will ... | ⬜ | ⬜ | ⬜ | ⬜ | ⬜ | ⬜ |
| The pressure on patients to consider assisted suicide will ... | ⬜ | ⬜ | ⬜ | ⬜ | ⬜ | ⬜ |
| The pressure on professionals to offer assisted suicide will ... | ⬜ | ⬜ | ⬜ | ⬜ | ⬜ | ⬜ |
| The tendency among professionals to actively end a patient's life, at the patient's request and with the aim of ending the patient's suffering, will ... | ⬜ | ⬜ | ⬜ | ⬜ | ⬜ | ⬜ |
| The tendency among professionals to end the life of a person who has lost his or her decision making capacity in order to end his or her suffering will ... | ⬜ | ⬜ | ⬜ | ⬜ | ⬜ | ⬜ |

#### 10. Do you think that patients should have the right to assisted suicide within:

|  | **yes** | **Yes, but with conditions** | **No** | **I don't know** |
| --- | --- | --- | --- | --- |
| Nursing homes | ⬜ | ⬜ | ⬜ | ⬜ |

From the following proposals, choose the one that suits you best:

- These conditions should be stricter than the current ones at HUG/CHUV
- These conditions should be less strict than the current ones at the HUG/CHUV
- These conditions should be identical to those currently in force at the HUG/CHUV
- I don't know

|  | **yes** | **Yes but with conditions** | **No** | **I don't know** |
| --- | --- | --- | --- | --- |
| Palliative care facilities or units | ⬜ | ⬜ | ⬜ | ⬜ |

From the following proposals, choose the one that suits you best:

- These conditions should be stricter than the current ones at HUG/CHUV
- These conditions should be less strict than the current ones at the HUG/CHUV
- These conditions should be identical to those currently in force at the HUG/CHUV
- I don't know

|  | **yes** | **Yes but with conditions** | **No** | **I don't know** |
| --- | --- | --- | --- | --- |
| Hospitals | ⬜ | ⬜ | ⬜ | ⬜ |

From the following proposals, choose the one that suits you best:

- These conditions should be stricter than the current ones at HUG/CHUV
- These conditions should be less strict than the current ones at the HUG/CHUV
- These conditions should be identical to those currently in force at the HUG/CHUV
- I don't know

**11. If the patient is facing a clinical situation that is almost impossible to manage outside your facility, you decide to**

*(One answer only)*

- arrange the logistics for a return home so that the assisted suicide can take place upon arrival
- Keep the patient in your care unit and the patient withdraws from assisted suicide
- you accommodate the patient's request for assisted suicide in your unit

**11 bis. If the person requesting assisted suicide is - from a medical point of view - able to return to his or her place of living to carry out the act**

Do you think this return should be?

- Mandatory
- Optional
- I don't know

**12. Would you consider assisted suicide for yourself?**

*Please select one answer only*

- Yes
- Rather yes
- Rather no
- No
- I don't know
- I prefer not to answer

**13. Do you think assisted suicide should be allowed in our hospital?**

Please select your answer

- Yes
- No
- I don't know

**14. If assisted suicide was allowed in our hospital, who should, in principle, be allowed to provide assisted suicide under proper legal regulation?**

Select all that apply

- Organizations providing assisted suicide
- Qualified in-house physicians
- Qualified physicians from outside the hospital
- In-house qualified nurses
- Qualified nurses from outside the hospital
- Others (please specify): _________________________________________________

**15. Which of the following actions do you consider to constitute assisted suicide?**

| Yes | No | I don't know |  |
| --- | --- | --- | --- |
| ⬜ | ⬜ | ⬜ | 1. Issue the prescription for a life-ending drug or hand over the drug |
| ⬜ | ⬜ | ⬜ | 1. Participate in the preparation of the suicide, e.g. by placing or leaving a venous line, or by giving specific indications for the suicide |
| ⬜ | ⬜ | ⬜ | 1. Be present at the moment of suicide until death occurs |
| ⬜ | ⬜ | ⬜ | 1. Refer the person to a physician who may be willing to provide assisted suicide |
| ⬜ | ⬜ | ⬜ | 1. Collaborate with an organization providing assisted suicide |
| ⬜ | ⬜ | ⬜ | 1. Collaborate with a person outside the hospital, such as the general practitioner who will assist the patient |
| ⬜ | ⬜ | ⬜ | 1. Contact a person in the institution for assisted suicide |
| ⬜ | ⬜ | ⬜ | 1. Continue to treat or care for the patient without participating in the suicide |

**16. To conclude, please allow us to ask for some personal and professional information**

**Are you?**

- a man
- a woman

**What is your age?**

- 20-29
- 30-39
- 40-49
- 50-59
- 60-69
- 70-79

**Where did you get your degree?**

- In Switzerland
- Abroad

**What is your profession?**

- Health care assistant
- Nurse
- Physician
- Physiotherapist
- Ergo therapist
- Psychologist
- Chaplain
- Social worker
- Other (specify): ..............................

**How long have you been professionally active (since graduation)?**

…………. years

**In which department do you work?**

*{List of departments}*

**In which service do you work?**

*{List of services}*

**What do you consider your religion?**

- Catholic
- Protestant
- Muslim
- Jewish
- Buddhist
- Hindu
- Other religion
- I have no religion
- I do not wish to answer this question
